# Prevention of CpG-Induced Pregnancy Disruption by Adoptive Transfer of *In Vitro*-Induced Regulatory T Cells

**DOI:** 10.1371/journal.pone.0094702

**Published:** 2014-04-08

**Authors:** Yi Lin, Xiaorui Liu, Bin Shan, Ji Wu, Surendra Sharma, Yun Sun

**Affiliations:** 1 International Peace Maternity and Child Health Hospital, School of Medicine, Shanghai Jiao Tong University, Shanghai, China; 2 Medical Sciences, Washington State University Spokane, Spokane, Washington, United States of America; 3 Key Laboratory for the Genetics of Developmental and Neuropsychiatric Disorders (Ministry of Education), Bio-X Institutes, Shanghai Jiao Tong University, Shanghai, China; 4 Department of Pediatrics, Women and Infants Hospital of Rhode Island, Warren Alpert Medical School of Brown University, Providence, Rhode Island, United States of America; 5 Department of Reproductive Medicine, Renji Hospital, School of Medicine, Shanghai Jiao Tong University, Shanghai, China; 6 Shanghai Key Laboratory for Assisted Reproduction and Reproductive Genetics, Shanghai, China; New York University, United States of America

## Abstract

**Objective:**

To elucidate the mechanism by which embryo-resorption and preterm birth were enhanced by pathogenic CpG motif and to develop a counter strategy for normal pregnancy outcome.

**Methods:**

This is an animal model-based study. In pregnant nonobese diabetic (NOD) mice and wild-type (WT) mice in the same strain background, an infection was mimicked by toll-like receptor 9 (TLR9) activation through CpG1826-injection. *In vivo* inactivation of IL-10 was performed to enhance pregnancy loss. Regulatory T cells induced by FTY720 *in vitro* from splenic CD4^+^CD25^−^Foxp3^−^ cells (iTreg cells) were transferred to improve pregnancy outcomes in NOD mice.

**Results:**

Embryo-resorption and preterm birth were readily induced by CpG1826 in NOD mice, but not in WT mice. However, inactivation of IL-10 using neutralizing antibody injections enhanced pregnancy loss in WT mice exposed to CpG, while adoptive transfer of iTreg cells increased decidual Foxp3^+^ Treg cells and IL-10^+^ cell number and rescued pregnancy.

**Conclusions:**

NOD mice are prone to abortion and preterm birth. This can be attributed to lacking Treg cells and insufficient IL-10 expression. Adoptive transfer of iTreg cells can rescue CpG-mediated pregnancy failure.

## Introduction

Mammalian Toll-like receptors (TLRs) such as TLR9 initiate immune responses to infection by recognizing microbial nucleic acids [1]. In some cases, systemic or intrauterine bacterial infection results in excessive production of hypomethylated CpG DNA motifs that are recognized by TLR9 [2]–[4]. In mammals, CpG motifs trigger strong polarized immune responses that impair pregnancy and result in embryo loss or preterm birth [4], [5].

Previous studies suggested that cytokine IL-10 might be a determinant for pregnancy success. LPS caused adverse pregnancy outcomes including increased embryo resorption and preterm birth in IL-10^-/-^ mice compared with their wild-type (WT) counterparts even at very low doses [6], [7], [8], and low doses of CpG displayed similar effects [4], [5]. Notably, NOD mice are known to be lower in both regulatory T cells (Treg cell) number [9] and IL-10^+^ cell number [10], and prone to pregnancy loss even without inflammatory challenge [5], [9].

It was found that CD4^+^CD25^−^ T cells can be converted to CD4^+^CD25^+^ cells in the presence of TGF-β [11]. In NOD mice and other murine models, commercially available FTY720, 2-amino-2-[2-(4-octylphenyl)ethyl]propane-1,3-diol, also named fingolimod, effectively converted conventional Foxp3^−^CD4^+^CD25^−^ cells into Foxp3^+^CD4^+^CD25^+^ cells (induced Treg cells, iTreg cells) *in vitro* and *in vivo* [12], [13]. When cultured in conditioned medium containing FTY720 (10 ng/mL), the percentage comprised by the fork head boxp3/winged helix-expressing (Foxp3^+^) subset within CD4^+^ T cells harvested from WT or NOD mice was increased, which probably was contributed to Foxp3-positive conversion from both CD4^+^CD25^+^ and CD4^+^CD25^−^ cells [12], [13]. However, it remains unclear whether adoptive transfer of these cells can effectively rescue pregnancy loss in NK-cell deficient NOD mice with insufficient IL-10 production [10].

Our recent study found that NOD mice were sensitive to CpG-mediated pregnancy loss, while WT counterparts are resistant to CpG stimulation [5]. However, the mechanism for this differential response is not fully elucidated. In this study, we examined the role of the CpG-TLR9 pathway during pregnancy in a mouse model with a focus on the role of Treg cells and IL-10 in protecting pregnancy [12], [13]. Our observations indicate a link between immune response mediated by CpG and immune tolerance mediated by Treg cells and IL-10 at the feto-maternal interface.

## Materials and Methods

### Mice and Ethics

Mice used in this study, wild-type (WT) female BALB/c, female NOD in the BALB/c background, and male C57BL/6 (8–12 weeks old), were purchased from the National Resource Center for Mutant Mice, Model Animal Research Center, Nanjing University (Nanjing, China). All mice were housed in a pathogen-free facility. The immunodeficiency of NOD mice was confirmed using methods described previously [14]–[17]. All animal procedures followed the national animal care guidelines, and were approved by Shanghai Jiao Tong University's Institutional Animal Care and Use Committee. All related data were approved for publication by Shanghai Jiao Tong University's Institutional Review Board and Ethics Committee. Each experimental group contained at least four mice. The day of vaginal plug appearance was designated as gestational day 0.5 (E0.5). All the reagents were purchased from BioLegend unless otherwise indicated.

### Injection of CpG1826 into Pregnant Mice

WT and NOD mice were injected intraperitoneally (IP) with CpG1826 (InvivoGen) at doses of 15, 25, 100, 300, 400, or 500 μg per dam on E6.5 in embryo-resorption experiments and E14.5 in preterm-birth experiments [4], [5]. In the embryo-resorption experiments, CpG1826 was administered on E6.5 and embryo resorption rate was measured on E9.5. Embryos with smaller size (< 20% of the average size), hemorrhage (at the implantation site), and necrosis were identified as resorbed embryos [18]. In the preterm birth experiments, CpG1826 injection was performed on E14.5 and mice were examined for signs of preterm birth of live pups (no less than 4 times a day at an interval of < 8 h) [19]. Preterm birth was defined as the finding of at least one pup in the cage or the lower vagina before E18.5 (not including E18.5) [19].

### Neutralizing anti-IL-10 Ab Treatment

**Figure 1:**
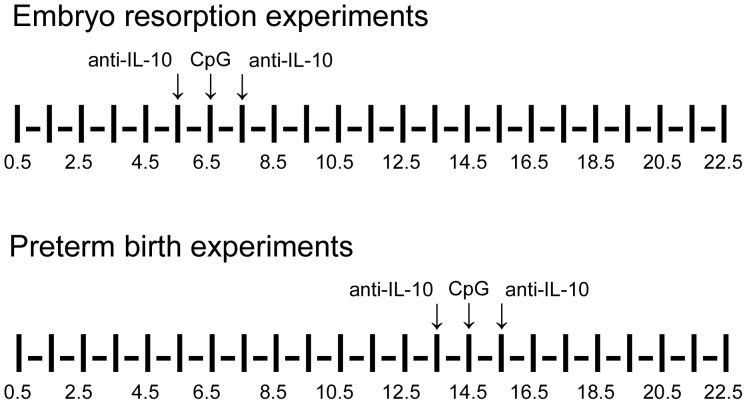
Description of methods for IL-10-inactivation and CpG-stimulation. Anti-mouse IL-10 Ab was administered IP into pregnant mice at a dose of 250 μg on E5.5 and E7.5 with CpG1826 injection on E6.5 in the embryo-resorption experiments, or on E13.5 and E15.5 with CpG1826 injection on E14.5. Arrows indicate the time-points when injections were performed.

Purified anti-mouse IL-10 Ab (catalog No. 504902) was administered IP dosing at 250 μg on E5.5 and E7.5 with CpG1826 injection on E6.5, or on E13.5 and E15.5 with CpG1826 injection on E14.5 (Fig. 1). Embryo-resorption and preterm birth was evaluated as described above. Placenta and decidual tissue were harvested for flow cytometric analysis [4], [5].

### Mononuclear and Granular Cell Preparation

Uterine mononuclear and granular cells (UMGCs) were isolated on E9.5 or at the indicated time points by mincing and mechanical dispersion of the whole uteroplacental tissue in RPMI1640 medium supplemented with 10% fetal bovine serum, penicillin/streptomycin, and L-glutamine. Single-cell suspensions from uterine horns were sifted through a 100-µm cell strainer followed by density gradient separation using Fico-Lite LM (Atlanta Biologicals). These experiments were performed on the three layers obtained from Ficoll gradient separation to determine in which layer granulocytes accumulated. It was found that granulocytes were concentrated directly below the monocyte layer. Both layers were harvested together for further experiments [4], [5].

### Purification of CD4^+^, CD4^+^CD25^+^, and CD4^+^CD25^−^ Cells

Splenic CD4^+^ cells were collected from virgin WT mice and purified by magnetic activated cell sorting or magnetic affinity cell sorting (MACS) using microbeads-conjugated anti-mouse CD4 mAb. The purified CD4^+^ cells were further separated into CD4^+^CD25^+^ and CD4^+^CD25^−^ subsets by MACS using microbeads-conjugated anti-mouse CD4 and anti-mouse CD25 mAbs (all from Miltenyi Biotech, Auburn, CA) following the manufacturer's instructions. The purity and viability of the purified cells routinely exceeded 97% and 95%, respectively, as determined by flow cytometry and propidium iodide staining (Invitrogen, Eugene, OR) [15], [20].

### Generation of Treg Cells Induced by FTY720

Treg cells were generated by *in-vitro* CD4^+^CD25^−^ cell induction using FTY720-containing RPMI 1640 medium [12], [13]. In brief, *in-vitro* culture system was developed for Treg cell generation using naive precursor CD4^+^CD25^−^ T cells isolated from NOD mice which have reduced Treg cell number [11]–[13], [21]. CD4^+^CD25^+^ cells and CD4^+^CD25^−^ cells were purified from NOD mice with the same method used in WT mice. The absence of Treg cells was confirmed first in NOD CD4^+^CD25^−^ T cells by flow cytometry. After RBC lysis and several washings, a total of 2×10^6^ cells were recovered and cultured in 1-mL volume with previously optimized doses of plate-bound anti-CD3 Ab (0.125 μg /mL in 200 μL volume), rIL-2 (25 U/ mL), and FTY720 (10 ng /mL) for 6 days at 37°C in a 5% CO_2_ incubator in 48-well plates. MACS-purified CD4^+^CD25^+^ cells from WT counterparts were also cultured under the same condition to induce Treg cells. In control groups, cells were cultured in the conditioned medium without FTY720. After 6 days, the phenotype of cells was characterized by flow cytometry [11]–[13], [21].

### Cell Sorting and Transfer

FTY720-induced CD4^+^CD25^+^ cells and CD4^+^CD25^−^ cells were i.v. transferred into pregnant mice (2×10^6^ cells for each mouse) 8 hours after CpG challenging on E6.5, and the embryo-resorption rate was measured on E9.5. In other cases, CpG challenging was performed on E14.5 and preterm birth was evaluated as described. Whole uteroplacental tissue was harvested for further analysis on Day 3 and Day 9 after adoptive transfer in embryo-resorption experiments [4], [5].

### Flow Cytometry

Abs specific for murine CD45 (clone: 30-F11), CD4 (L3T4), CD25 (PC61), Foxp3 (3G3), and IL-10 (JES5-16E3) were purchased from BioLegend. Isolated UMGCs were washed in phosphate-buffered saline (PBS) and resuspended in PBS containing 2% FBS (staining buffer). For extracellular staining, the cells were incubated in the indicated combinations of Abs for 30 minutes on ice, rinsed with staining buffer, and assayed on a FACS Calibur flow cytometer using CellQuest software (BD Biosciences). Isotype controls were established by staining of isotype control Abs to exclude false-positive cells [18], [22]. Abs specific for Foxp3 and IL-10 were purchased for intracellular staining. UMGCs were washed with staining buffer and incubated in 96-well plates for 4–6 hours with Brefeldin A (BD Biosciences), PMA (Calbiochem), and ionomycin (Calbiochem). Cells were washed twice with staining buffer and stained for cell surface antigens as described above. For staining of intracellular antigens, UMGCs were washed with Perm Wash (BD Biosciences) and fixed with Cytofix/Cytoperm (BD Biosciences) for 25 minutes on ice and incubated with Abs for 30 minutes at room temperature. Cells were washed and analyzed using flow cytometry. Experiments were performed independently 4 times, and data were shown as mean±SD [4], [5], [23].

### Statistical Analysis

Embryo resorption rate was compared among the groups using χ^2^ test. Flow cytometry data were analyzed using Quad statistics. An ANOVA was firstly used to show the effects of treatments in experiments where multiple groups were compared, and Student's *t* test was used as a post-hoc test. Experiments in flow cytometry were conducted independently four times in each group and the results were given as mean±SD [23]–[25].

## Results

### CpG ODN Significantly Increased Pregnant Loss in NOD Mice, But Not in WT Counterparts

**Figure 2:**
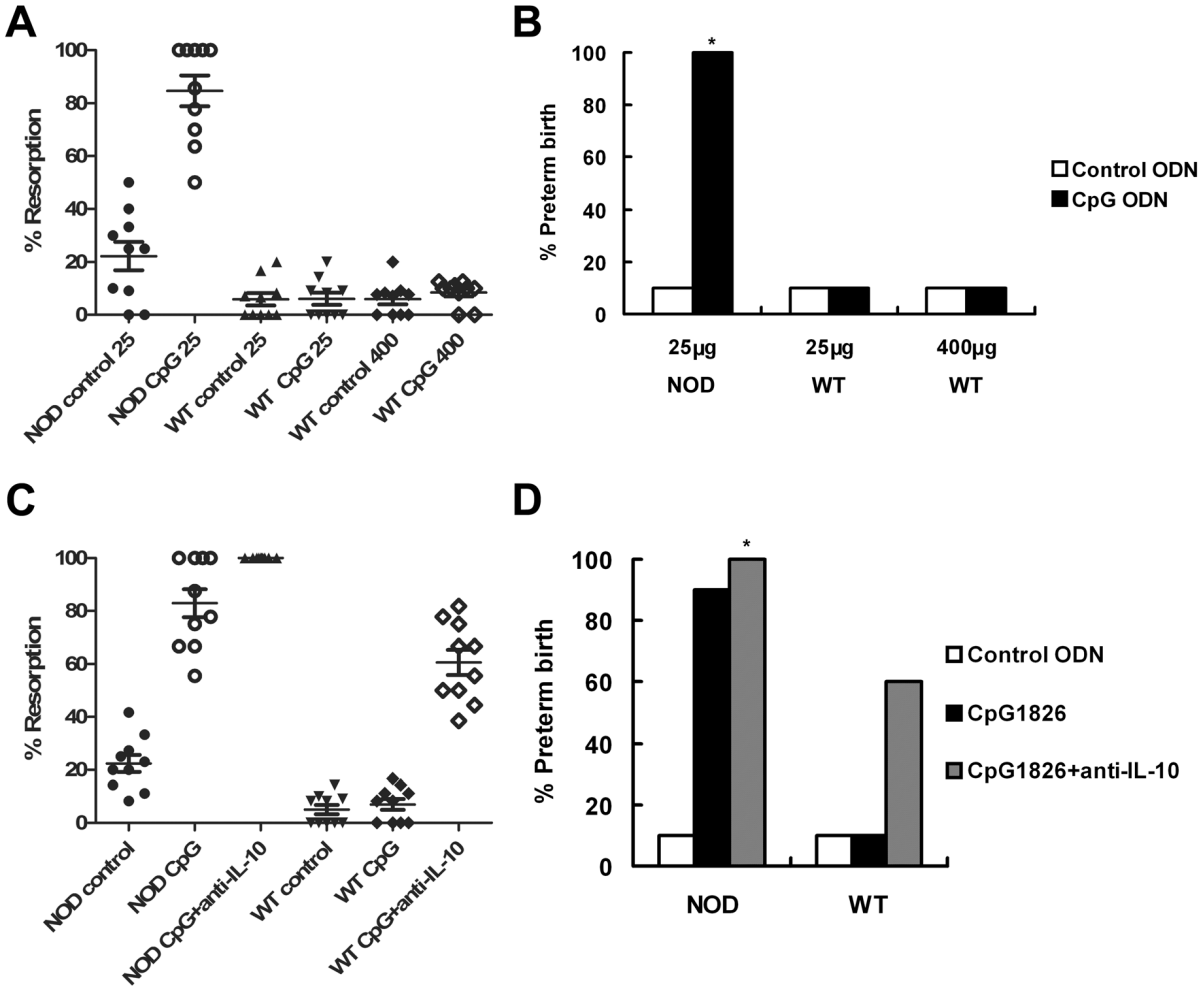
CpG-induced increase of fetal resorption and preterm birth in NOD mice. CpG1826 or control ODN was injected IP at the indicated doses (25 μg/dam for NOD mice, and 25 or 400 μg/dam for WT mice). (A) Mice were injected with CpG or control ODN at E6.5 and fetal resorption was assessed at E9.5. (B) Injection was performed at E14.5, and the preterm birth rate was assessed. (C) In addition to CpG or control ODN injection (25 μg/dam), anti-IL-10 neutralizing Ab was injected one day after CpG or control ODN injection, and fetal resorption was assessed at E9.5. (D) Anti-IL-10 Ab injection was performed one day after CpG or control ODN injection (25 μg/dam), and preterm birth was assessed. * Maximum preterm birth rate (100%) was observed at the indicated dose of CpG. Ten female mice were used in each group.

To evaluate the ability of CpG1826 to induce embryo resorption, doses ranging from 15 to 500 μg/dam were used. A significantly higher embryo-resorption rate was found in NOD mice than in WT mice even under the condition where only control ODN was administered (24.3% [25 of 103] vs. 5.7% [7 of 122]). The 25 μg/dam dose of CpG1826 was sufficient to induce significantly increased embryo-resorption in NOD mice when evaluated at gestational day 9.5 (E9.5). At this dose, CpG1826 increased embryo resorption rate in NOD mice (82.8% [77 of 93]; *P*<0.01 vs. control ODN-treated NOD mice), but not in WT counterparts (6.4% [7 of 110]) (Fig. 2A). Maternal wasting was also observed in NOD mice when CpG was given at a dose of 100 μg/dam or higher, and in WT mice when CpG was given at a dose of 500 μg/dam [4], [5]. No cranial or distal limb malformation was found in pups.

A similar trend was observed in preterm birth experiments. NOD mice more frequently delivered pups at the dose of 25 μg/dam within 72 hours of injection than control ODN-treated mice (100% vs. 10% in control ODN-treated NOD mice). No negative pregnancy outcome was observed after CpG1826-treatment in WT mice at the same dose or higher doses up to 400 μg/dam (Fig. 2B), and no malformation was found in the delivered pups.

### Inactivation of IL-10 Increased Pregnancy Loss in CpG-Induced WT Mice

A neutralizing Ab was used for *in vivo* inactivation of IL-10. Though WT mice resisted to CpG-induced pregnancy loss at a high dose of 400 μg/dam, both embryo-resorption (61.4% [51 of 83] vs. 7.3% [8 of 109]; *P*<0.01) and preterm birth (60% vs. 10%; *P*<0.05) rates were increased in these mice when neutralizing anti-IL-10 Ab was injected together with CpG ODN stimulation as indicated. In comparison, anti-IL-10 treatment caused only a modest increase in the embryo-resorption rate and preterm birth rate in CpG-induced NOD mice and the increase was not statistically significant (embryo resorption: from 81.7% [58 of 71] to 100% [72 of 72]; preterm birth: from 90% [9 of 10] to 100% [10 of 10]) (Fig. 2, C and D).

As shown in Fig. 2B, CpG-injection in NOD mice results in 100% [10 of 10] preterm birth rate, while in another experiment, CpG-injection at the same dose in NOD mice results in 90% [9 of 10] preterm birth rate (Fig. 2D). However, no statistically supported significance was found between these two rates in preterm birth.

### FTY720 Potently Induced Foxp3^+^ Treg Cell Conversion from CD4^+^CD25^−^Foxp3^−^ Cells

**Figure 3:**
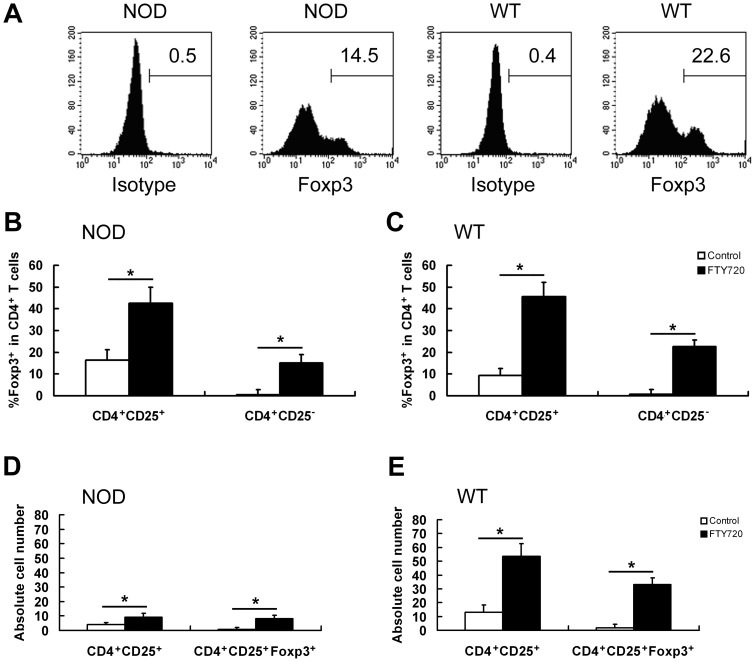
Generation of Foxp3^+^ Treg cells from conventional splenic cells induced by FTY720. The percentage of Foxp3^+^ Treg cells was significantly increased upon FTY720 induction *in vitro*. CD4^+^CD25^+^ and CD4^+^CD25^−^ cells were purified as indicated and cultured in the presence of FTY720 to generate Foxp3^+^ Treg cells from CD4^+^CD25^−^ cells or increase the percentage of Foxp3^+^ cells in CD4^+^CD25^+^ cells. Foxp3 expression was measured using flow cytometry. (A) Representative results in flow cytometry. CD4^+^ cells were gated. A considerable proportion of Foxp3^+^ cells were found in FTY720-induced CD4^+^CD25^−^ cells from both NOD and WT mice. (B and C) Data summary of flow cytometry. The percentage of Foxp3^+^ subset of CD4^+^ cells was significantly increased in both FTY720-induced CD4^+^CD25^+^ cells and FTY720-induced CD4^+^CD25^−^ cells. The same trend was observed in both NOD and WT mice. (D and E) Absolute number of indicated cells with or without FTY720-induction in MACS-purified CD4^+^CD25^+^ cells (×10^5^). * *P*<0.01. Experiments were conducted independently for 4 times.

In FTY720-containing RPMI 1640 medium, the cultured CD4^+^CD25^−^Foxp3^−^ splenocytes from NOD or WT mice were converted into Foxp3^+^ Treg cells (iTreg cells) (Fig. 3A). Similar results were observed in CD4^+^CD25^+^ cells.

In NOD mice, the percentage of Foxp3^+^ subset in CD4^+^ cells was 15.1%±3.5% after the conventional CD4^+^CD25^−^ cells were cultured for 6 days in the FTY720-containing medium. The percentage was 42.5%±7.5% after CD4^+^CD25^+^ cells were cultured under the same condition. (Fig. 3, B and D)

In WT mice, the percentage of Foxp3^+^ subset in CD4^+^ cells was 22.6%±4.6% after the conventional CD4^+^CD25^−^ cells were cultured for 6 days in the FTY720-containing medium. The percentage was increased from 9.3%±3.2% to 45.7%±6.4% after CD4^+^CD25^+^ cells were cultured under the same condition (Fig. 3, C and E).

### Effects of iTreg Cell Transfer on Pregnancy Outcomes

**Figure 4:**
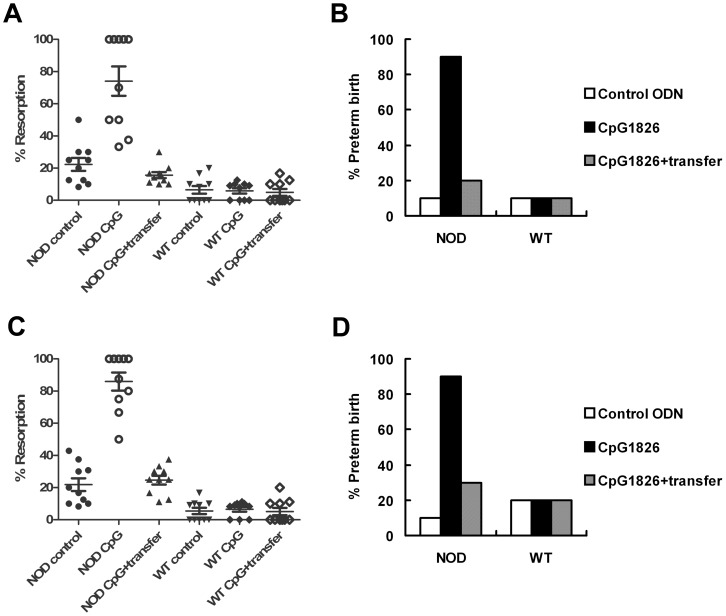
Effect of iTreg cell transfer on CpG-induced embryo resorption and preterm birth. (A and B) Adoptive transfer of iTreg cells generated from CD4^+^CD25^+^ cells. (C and D) Adoptive transfer of iTreg cells generated from CD4^+^CD25^−^ cells. Both kinds of transferred cells efficiently abrogated the CpG-induced increase of embryo-resorption and preterm birth in NOD mice. No change of embryo-resorption or preterm birth was observed in WT mice treated with CpG or cell transfer. Ten female mice were used in each group.

CD4^+^CD25^−^ cells are mainly composed of naïve T cells, while iTreg cells are included in the CD4^+^CD25^+^ cell subset. When the iTreg cells generated from both CD4^+^CD25^+^ cells (Fig. 4, A and B) and CD4^+^CD25^−^ cells ^-^ (Fig. 4, C and D) were transferred into pregnant mice, respectively, the CpG1826-induced increase of fetal resorption and preterm birth in NOD mice were drastic abrogated. But no significant change of the embryo-resorption or preterm birth rate was observed in WT mice.

Because the iTreg cells from NOD mice and WT mice were similar in their capacity to prevent pregnancy loss, the cells used for adoptive transfer were mainly purified from WT mice in our further pregnancy rescue experiments.

### Effects of iTreg Cell Transfer on Decidual Foxp3^+^ Cell Number

**Figure 5:**
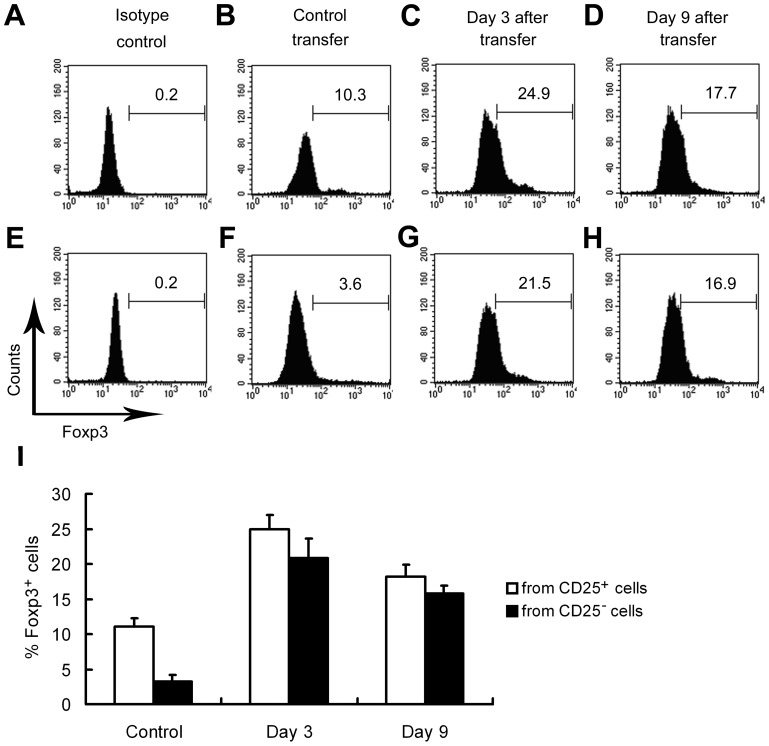
Effect of iTreg cell transfer on decidual Foxp3^+^ cell number. UMGCs were harvested after adoptive transfer of iTreg cells. Foxp3^+^ iTreg cells from WT splenic CD4^+^CD25^+^ T cells were generated by FTY720-induction (named as ‘from CD25^+^ cells' group) (A-D), or from conventional WT splenic CD4^+^CD25^−^ T cells (named as ‘from CD25^−^ cells' group) (E-H). In the ‘control transfer' group, control cells without FTY720-induction were transferred. UMGCs were harvested and analyzed on Day 3 after transfer. The percentage of decidual Foxp3^+^ subset of CD4^+^ cells was indicated. (I) Data summary of flow cytometry. * *P*<0.05 vs. Day 3 and Day 9 FTY720-induced cell transfer group.

Decidual Foxp3^+^ cells were analyzed using flow cytometry after cell transfer of natural CD4^+^CD25^+^ (control cells) and FTY720-induced CD4^+^CD25^−^ cells (iTreg cells), respectively. As shown in Fig. 5, when the control cells without FTY720-induction were transferred, the percentage of decidual Foxp3^+^ subset in CD4^+^ population was approximately 11% (11.1%±0.8%). However, when FTY720-induced cells were transferred, the percentage of decidual Foxp3^+^ subset in CD4^+^ cells on Day 3 and Day 9 after transfer was approximately 2.2-fold (24.9%±2.3%) and 1.6-fold (18.2%±1.9%) as high as the control group in which only control cells were transferred (*P*<0.01).

### Effects of iTreg Cell Transfer on Decidual IL-10^+^ Cell Number

**Figure 6:**
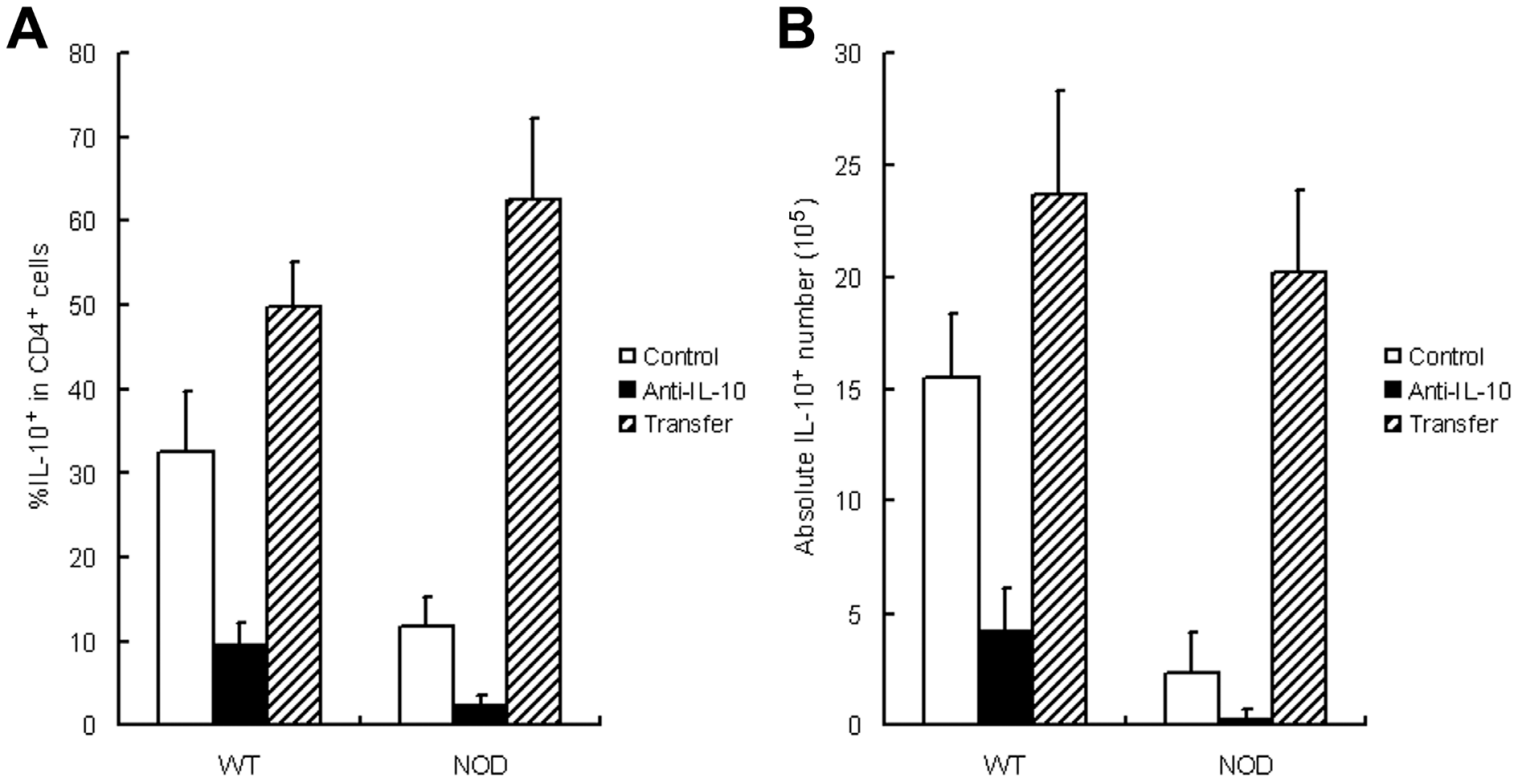
Effect of iTreg cell transfer on decidual IL-10^+^ cell number. UMGCs were harvested after adoptive transfer of iTreg cells. The relative (A) and absolute (B) IL-10^+^ cell number was measured using flow cytometry after anti-IL-10 Ab injection and iTreg cell transfer. The transferred cells were derived from FTY720-induction on conventional CD4^+^CD25^−^ cells.

The percentage of decidual IL-10^+^ subset in CD4^+^ cell population was also evaluated by flow cytometry after anti-IL-10 Ab treatment or adoptive transfer of the iTreg cells from WT splenic CD4^+^CD25^+^ cells (data not shown) or CD4^+^CD25^−^ cells induced by FTY720 (Fig. 6A). In NOD mice, IL-10^+^ percentage decreased after anti-IL-10 treatment (from 11.7%±3.3% to 2.2%±0.8%), but increased on Day 3 after cell transfer (62.5%±9.6%; *P*<0.01 for both). A similar trend was observed in WT mice (from 32.5%±7.2% to 9.5%±4.3% after anti-IL-10 treatment, but increased to 49.7%±5.9% after cell transfer; *P*<0.01 for both) (Fig. 6A).

In NOD mouse, the absolute number of IL-10^+^ cells also significantly decreased after anti-IL-10 treatment, but increased after adoptive transfer of the iTreg cells from WT splenic CD4^+^CD25^−^ cells (from 2.3×10^5^ to 0.3×10^5^ cells after anti-IL-10 treatment, but increased to 20.2×10^5^ cells on Day 3 after cell transfer; *P*<0.01 for both). A similar trend was observed in WT mice (from 15.5×10^5^ to 4.2×10^5^ cells after anti-IL-10 treatment, but increased to 23.7×10^5^ cells after cell transfer; *P*<0.01 for both) (Fig. 6B).

## Discussion

Previous research has shown that murine models of NK cell deficiency, such as NOD mice, are prone to spontaneous embryo loss and infertility. Restoring NK cell function improves pregnancy outcomes in NOD mice [9]. Conversely, depletion of NK cells increased embryo loss in NOD/SCID mice that retained remnants of functional NK cell populations [15], [16], [26]. In addition, when NK cell function was restored in NOD mice, embryo-resorption rates widely varied in mice [9]. We could separate these mice into the higher and lower embryo-resorption groups, and the higher embryo-resorption NOD mice had lower Foxp3^+^ T cell number [9].

Both T and NK cells are present in the pregnant uterus and are thought to play a critical role in modulation of maternal tolerance to an allogeneic fetus [27]–[31]. NOD mice suffer from impaired NK cell function and possibly the cross-talk between T cells and NK cells is impaired. Thus, NOD mice are prone to T cell-mediated diabetes based on T cell-mediated immune attack on pancreas β-cells [32]. In pregnant NOD mice, imbalance of T cell and NK cell function in the gestational tissue microenvironment may be related to increased pregnancy loss [11]–[13].

Though NOD mice are prone to pregnancy loss, the absolute number of pregnancy loss rate in natural pregnant NOD mice is only slightly but statistically higher than WT mice. In contrast, the pregnancy loss rate in NOD mice is remarkably higher when CpG challenge or anti-IL-10 injection is performed, as compared with the WT mice with the same treatment. Thus, we select a CpG-challenging and an IL-10-inhibiting model established in the NOD mice.

Treg cells express CD25 and a signature marker, Foxp3, and play a critical role in allo-immune tolerance induction [33]. The characteristic phenotype of Treg cells is Foxp3^+^CD4^+^CD25^+^ which can be detected on these cells naturally derived from thymus or derived extrathymically upon peripheral stimulation [28], [33]. Therefore, adoptive transfer of Treg cells may be a promising therapy to abortion and preterm birth in Treg cell-deficient subjects.

FTY720 has been demonstrated to bear therapeutic potential in autoimmune and allergic disorders and suppress graft-vs-host response in transplant rejection [34]. Upon phosphorylation, FTY720 is known to mimic the action of sphingosine-1-phosphate (S1P) and acts as an agonist of four of five S1P receptors [35]. When binding to S1P receptors on lymphocytes, FTY720 alters trafficking patterns of lymphocytes as migration of CD4^+^ T-cell into lymph nodes is elevated and their egress is impeded [36]. Other studies found that Foxp3^+^CD4^+^CD25^+^ cells are generated from conventional CD4^+^CD25^−^Foxp3^−^ cells when cultured in the conditioned-medium containing FTY720 [11]–[13], [21]. The function of these converted cells remains largely unknown.

In this study, FTY720 is used to induce CD4^+^CD25^−^ cell conversion into Foxp3^+^ cells in order to obtain more Foxp3^+^ cells. These Foxp3^+^ cells are used to prevent pregnancy loss in NOD mice challenged with CpG1826. After FTY720-treatment, the percentage of Foxp3^+^ cell subset is increased in both CD4^+^CD25^+^ and CD4^+^CD25^−^ cell subsets. However, the conversion rate of induced cells converted into Foxp3^+^ cells is significantly higher in FTY720-treated CD4^+^CD25^−^ cells than FTY720-treated CD4^+^CD25^+^ cells (Fig. 3).

Consequently, Foxp3^+^CD4^+^CD25^+^ cells from CD4^+^CD25^−^Foxp3^−^ cells by FTY720-induced conversion were adoptively transferred into abortion-prone NOD mice [11]–[13], [21]. Both embryo-resorption and preterm birth rates were significantly decreased after cell transfer. In addition, adoptive transfer of these cells rescued the pregnancy loss induced by IL-10 inactivation in WT mice. The findings suggest that adoptive transfer of these cells may be a promising strategy in prevention of abortion and preterm delivery, especially those resulted from CpG-stimulation or similar challenges.

We previously found that NOD mice are prone to low-dose CpG stimulation and to CpG-mediated embryo-resorption and preterm birth. In contrast, WT counterparts are resistant to CpG stimulation at the same dose and even a higher dose [4], [5]. However, the mechanism was not fully elucidated. In addition, our previous work found that the percentage of IL-10^+^ cells in the decidual CD45^+^ cell population derived from NOD mice was significantly lower than WT mice [10]. NK cells have been shown to produce IL-10 in response to viral infection [37]. It is possible that NK cells from NOD mice are deficient in IL-10 production and this contributes to an inflammatory milieu. Thus NOD mice may respond to CpG in a manner similar to that in IL-10^-/-^ mice. The findings in this study are in strong agreement with those observed in IL-10^-/-^ mice [4].

To inhibit inflammatory pathologies, IL-10 functions at different stages of immune response and different anatomical locations. IL-10 was initially described as a T helper 2 (Th2)-type cytokine [38], but further evidence indicated that IL-10 production was associated with tolerant or Treg cell responses [39].

IL-10 is a much more broadly expressed cytokine by many cells in the adaptive immune system and innate immune system [40], [41]. The function and expression pattern of IL-10 in feto-maternal microenvironment may be distinct to that in extra-uterine tissues, because immune cells in decidua have unique phenotypes, functions, and constituent ratio [15], [42]. In this study, *in vivo* inactivation of IL-10 by its specific Ab significantly increased both embryo-resorption and preterm birth rates in WT mice that are resistant to CpG-mediated pregnancy loss when challenged at certain doses. In contrast, adoptive transfer of CD4^+^CD25^–^-converted Treg cells significantly increased the proportion and absolute number of decidual IL-10^+^ cells and prevented pregnancy loss. These findings indicate that IL-10 is critical to keeping a beneficial feto-maternal microenvironment when mammals are challenged by CpG or other pathogens.

FTY720 is believed capable of modulating peripheral effector and regulatory T cells in multiple sclerosis patients and proven to be effective in the treatment on this disease [43], [44]. Our findings suggest that adoptive transfer of FTY720-induced cells are effective in preventing CpG-induced embryo loss and preterm birth in NOD mice, and that FTY720 may be useful in developing a non-toxic therapy for reproductive failures.
